# Evaluation of Four Point of Care (POC) Antigen Assays for the Detection of the SARS-CoV-2 Variant Omicron

**DOI:** 10.1128/spectrum.01025-22

**Published:** 2022-05-26

**Authors:** Justin Hardick, Nicholas Gallagher, Jaiprasath Sachithanandham, Amary Fall, Zishan Siddiqui, Andrew Pekosz, Yukari C. Manabe, Heba H. Mostafa

**Affiliations:** a Department of Medicine, Division of Infectious Diseases, School of Medicine, Johns Hopkins University grid.471401.7grid.21107.35grid.471401.7 , Baltimore, Maryland, USA; b Department of Pathology, Division of Medical Microbiology, School of Medicine, Johns Hopkins University grid.471401.7grid.21107.35grid.471401.7 , Baltimore, Maryland, USA; c Bloomberg School of Public Health, Johns Hopkins University grid.471401.7grid.21107.35grid.471401.7 , Baltimore, Maryland, USA; d Department of Medicine, Division of Hospital Medicine, School of Medicine, Johns Hopkins University grid.471401.7grid.21107.35grid.471401.7 , Baltimore, Maryland, USA; University of Arizona/Banner Health

**Keywords:** Omicron variant, rapid antigen tests, point-of-care, limit of detection, LOD, ddPCR, SARS-CoV-2

## Abstract

Ensuring SARS-CoV-2 diagnostics that can reliably detect emerging variants has been an ongoing challenge. Due to the rapid spread of the Omicron variant, point-of-care (POC) antigen tests have become more widely used. This study aimed at (i) comparing the analytical sensitivity (LOD) of 4 POC antigen assays, BD Veritor, Abbott BinaxNow, Orasure InteliSwab and Quidel QuickVue, for the Omicron versus the Delta variant and (ii) verifying the reproducible detection of Omicron by the 4 antigen assays. The LOD for all four assays were evaluated using Omicron and Delta virus stocks quantified for infectivity and genome copies. The four assays detected all replicates of Omicron and Delta dilutions at 10^4^ and 10^5^ TCID50/mL, respectively. We quantified both viral stocks using droplet digital PCR (ddPCR), which revealed that the Omicron stock had equivalent copies of the N gene to Delta at a one log lower infectious virus. The Abbott BinaxNow and Orasure InteliSwab had the highest analytical sensitivity for Omicron while the Orasure InteliSwab and the Quidel QuickVue had the highest analytical sensitivity for Delta. When 14 SARS-CoV-2 real-time PCR positive nasal/nasopharyngeal swab samples (12 Omicron and 2 Delta, mean Ct = 19.1), were tested by the four assays, only the QuickVue detected all samples. Antigen test positivity correlated with recovery of infectious virus on cell culture in 9 out of 13 tested specimens from symptomatic, asymptomatic, unvaccinated, and vaccinated individuals. Although our study confirms the reduced analytical sensitivity of antigen testing compared to molecular methods, the Omicron variant was detectable by the four evaluated rapid antigen tests.

**IMPORTANCE** In the manuscript, we report an evaluation of the capability of 4 point of care (POC) antigen assays, the BD Veritor, Abbott BinaxNow, Orasure InteliSwab and Quidel QuickVue to detect the Omicron variant of SARS-CoV-2, and we compared their analytical sensitivity for Omicron versus Delta. In this analysis we found that all four assays detected Omicron and Delta at 10^4^ and 10^5^ TCID50/mL, respectively. We further quantified the viral stocks used by droplet digital (ddPCR) and found that the Omicron stock had equivalent copies of the N gene to Delta at a one log lower infectious virus titer and that an increased RNA to infectious virus ratio may be contributing to discrepancies in limit of detection in Omicron compared to Delta. We evaluated 14 SARS-CoV-2 real-time PCR positive nasal/nasopharyngeal swab samples (12 Omicron and 2 Delta), with an average cycle threshold value of 19.1, and only the QuickVue showed 100% agreement.

## INTRODUCTION

Since the beginning of the SARS-CoV-2 pandemic, the importance of accurate diagnostic assays has been highlighted (1). As new variants of SARS-CoV-2 have emerged, both molecular and immunologic diagnostic assays have been repeatedly evaluated to ensure sensitive and accurate detection of emerging SARS-CoV-2 variants (2–5). Although the utility of POC rapid antigen assays has been debated since the beginning of the pandemic, antigen tests were hypothesized to correlate with recovery of infectious virus on cell culture (6–9). With the emergence of the highly transmissible Omicron variant, POC rapid antigen tests could be a valuable tool to limit viral spread by expanding testing and reducing the time to results (10, 11). As such, it is important that POC rapid antigen assays are assessed for their capability to detect the Omicron variant. In this report, we evaluated four commonly used POC SARS-CoV-2 antigen assays that detect the nucleocapsid antigen (the BD Veritor (12), Abbott BinaxNow (13), Orasure InteliSwab (14), and Quidel QuickVue (15)) for their analytical sensitivity to Omicron versus Delta and the detection of Omicron from clinical swab samples.

## RESULTS

For LOD determination, all assays detected Delta replicates at 1 × 10^5^ TCID50/mL. For the Omicron variant, all assays were able to detect all replicates at 1 × 10^4^ TCID50/mL (Table 1). Omicron and Delta stocks were also quantified by ddPCR. When normalized to infectious virus titer, there was approximately 10-fold more viral RNA in the Omicron stocks compared to the Delta stocks (Table 1). Overall, the LOD assessment indicated that for both Delta and Omicron, the analytical sensitivity of the four antigen tests lies between 1.5 × 10^6^ and 1.5 × 10^7^ genome copies/mL with InteliSwab showing the highest analytical sensitivity for both variants combined (Table 1). Table 1 indicates the lowest concentration tested which was detectable by all assays evaluated.

**TABLE 1:** Analytical sensitivity of evaluated antigen assays for both Delta and Omicron

Variant	Delta		Omicron	
Concentration (TCID50/mL)	10^5^	10^4^	10^4^	10^3^
Concentration (Copies/mL)	1.5 × 10^7^	1.5 × 10^6^	1.5 × 10^7^	1.5 × 10^6^
BD Veritor	3/3^*a*^	0/4	3/3	0/4
Abbott BinaxNow	3/3	1/4	3/3	4/4
Orasure InteliSwab	3/3	3/3	3/3	3/3
Quidel QuickVue	3/3	3/3	3/3	1/4

a Positive replicates per total replicates tested.

For the clinical sample evaluation, Veritor detected 83% (10/12), BinaxNow and InteliSwab detected 92% (11/12), and QuickVue detected 100% (12/12) of Omicron positives (Table 2). The 12 tested Omicron samples’ N genes were similar based on sequence analysis (Table S1). The two Delta samples were used as controls where Quickvue was the only assay that detected the lineage AY.119 sample, and all assays detected the AY.25 sample (Table 2) (notably, there is an insertion in the Delta N protein sequences that are not in the Omicron N protein). Cell culture on VERO-TMPRSS2 cells revealed that 7 of 12 Omicron clinical samples had infectious virus; of which samples 4 and 11 were consistently missed by most of the evaluated assays. Sample 4 was collected 7 days after the onset of symptoms with no detectable anti-SARS-CoV-2 specific IgG antibodies (Table 2).

**TABLE 2:** Clinical samples used for the antigen assays’ evaluation

Sample	Ct^*a*^	Lineage (variant)^*b*^	Veritor^*c*^	BinaxNow^*d*^	InteliSwab^*e*^	QuickVue^*f*^	IgG^*g*^	Culture^*h*^	Symptoms (day^*i*^)	Status^*j*^ (type^*k*^)
1	18.81	BA.1.17 (Omicron)	+	+	+	+	NA^*l*^	5	No	Unvaccinated
2	19.02	BA.1.1 (Omicron)	+	+	+	+	+	−	No	Booster (Pfizer)
3	18.8	BA.1.15 (Omicron)	+	+	+	+	−	5	Yes	Full (Moderna)
4	18.81	BA.1.1 (Omicron)	−	−	+	+	−	4	Yes (7)	Full (Pfizer)
5	18.31	BA.1.1.16 (Omicron)	+	+	+	+	−	4	No	Full (Pfizer)
6	18.61	BA.1 (Omicron)	+	+	+	+	NA	−	No	Unvaccinated
7	18.29	BA.1.1 (Omicron)	+	+	+	+	−	4	No	Full (Pfizer)
8	19.1	BA.1.17 (Omicron)	+	+	+	+	−	6	Yes (1)	Full (Pfizer)
9	19.2	BA.1.1 (Omicron)	+	+	+	+	−	4	Yes (2)	Full (Moderna)
10	19.51	BA.1.1 (Omicron)	+	+	+	+	+	−	Yes (3)	Full (Pfizer)
11	19.71	AY.119 (Delta)	−	−/+ (very faint)	−/+ (very faint)	+	NA	3	Yes (0)	Unvaccinated
12	19.74	BA.1.1 (Omicron)	+	+	+	+	NA	NA	Yes (3)	Full (Pfizer)
13	19.89	AY.25 (Delta)	+	+	+	+	−	3	No	Booster (Moderna)
14	19.91	BA.1 (Omicron)	−	+	−	+	−	−	Yes (3)	Full (Pfizer)

a N gene Ct, cycle threshold.

b Lineage and variant identification of SARS-CoV-2 based on sequencing.

c Becton Dickinson Veritor assay.

d Abbott Molecular BinaxNow assay.

e Orasure InteliSwab assay.

f Quidel Quickvue assay.

g Detectable IgG SARS-CoV-2 antibodies.

h Cell culture day positive for SARS-CoV-2.

i Number of days since onset of symptoms.

j Individual SARS-CoV-2 vaccination status as either fully vaccinate, received booster or unvaccinated.

k SARS-CoV-2 vaccine manufacturer, either Pfizer or Moderna for this cohort.

l NA, not available.

## DISCUSSION

Our data indicate that the lower clinical sensitivity of some POC tests is not associated with the recovery of infectious virus, or antibody levels in the upper respiratory samples and are likely related to the analytical sensitivity of the assays for these particular variants. Our LOD results were similar to other studies (3, 16) illustrating a trend toward higher analytical sensitivity for the Omicron variant for the assays evaluated. However, concentrations of Delta and Omicron as determined by ddPCR indicated higher concentrations of Omicron genomes per infectious unit compared to Delta, which may contribute to the discrepancies in the sensitivity of antigen tests if materials used are only quantified in infectious units. Omicron has been shown to have enhanced replication in certain cell types (17, 18) but does replicate more slowly in others. An awareness of an altered genome to infectious virus ratio for Omicron is important when considering the relative sensitivity of antigen versus nucleic acid based diagnostic tests.

For our clinical sample evaluation, our results are similar to other studies (3, 4) particularly for PCR positive clinical samples with Ct values ≤30 and indicate that the assays evaluated are capable of detecting Omicron, although positive detections will rely on sufficient quantities of virus.

This study has limitations, notably the small clinical samples size evaluated (N = 14), and the relatively high concentration of virus within the clinical samples evaluated (mean N Ct =19.12). However, for our study, lower Ct value clinical samples were intentionally chosen to assess the performance of each assay for the detection of Omicron without the added variable of assay sensitivity associated with lower viral load. Additional limitations include the use of simulated anterior nasal swab samples for testing spiked and clinical specimens; even though this is a nonvalidated approach for sample collection and testing, most of our tested samples were positive and all the runs were valid (based on the assays’ internal control results) endorsing the validity of this approach. Lastly, LOD comparison studies were performed using relatively limited number of replicates for each tested concentration.

Our data confirm that antigen tests have lower sensitivity than nucleic-acid amplification tests. The four antigen tests that we evaluated were similar in their ability to detect Omicron variant. Antigen assays had a lower LOD with the Omicron variant which correlated with the higher number of nucleocapsid genomic copies per infectious virus concentration compared to Delta. Evaluations of new variants should include quantification of infectious virus and genomic viral RNA copies for comparing sensitivities of diagnostic tests.

## MATERIALS AND METHODS

### Limit of detection.

We performed a limit of detection (LOD) study for each assay with Delta and Omicron SARS-CoV-2 viral stocks that were quantified for infectious virus concentration by a tissue culture infectious dose (TCID50) assay and viral genome copy number ddPCR (Table 1). Delta and Omicron viral stocks were isolated and propagated by our team from positive clinical samples. SARS-CoV-2 cell culture and Bio-Rad SARS-CoV-2 ddPCR (19) were performed as previously described (20, 21).

### Clinical samples and clinical sample characteristics.

Nasal/nasopharyngeal (N = 14) samples with Ct values < 20 were selected from SARS-CoV-2 positive samples identified as Omicron at the Johns Hopkins Virology Laboratory as a part of whole-genome sequencing for surveillance in December 2021. The clinical diagnosis was performed by the NeuMoDx SARS-CoV-2 assay (22) and the samples mean N gene Ct was 19.12 (range 18.29 to 19.91). Variants’ lineages included (23) Omicron: BA.1 = 12, Delta: AY.119 = 1 and AY.25 = 1, (Table 2). Overall, 79% (11/14) of the patients were vaccinated with 81% (9/11) fully vaccinated based on CDC definitions. The majority, 73% (8/11), received the Pfizer vaccine (Pfizer, New York, NY), while 27% (3/11) received the Moderna vaccine (Moderna, Cambridge, MA). The majority (57%; 8/14) were symptomatic with a mean time from symptom onset of 2.3 days (range = 0 to 7 days). The clinical cohort data used for the evaluation is summarized in Table 2. Samples were collected under IRB approved protocol (IRB00288258), and sequencing was performed as previously described (23–26).

### Antigen testing.

Viral stock dilutions and clinical specimens were tested by each POC antigen test as per manufacturer instructions with a modification where the provided swab in the assay was immersed in each spiked or clinical specimen.

### Serology.

Serology was performed with the EUROIMMUN Anti-SARS-CoV-2 ELISA (IgG) following the package insert (27) on undiluted respiratory samples as described previously (25, 28).

### Viruses.

The Delta (SCV2/USA/MD-HP05660/2021; GISAID: EPI_ISL_2331507) and Omicron (SCV2/USA/MD-HP20874/2021; GISAID: EPI_ISL_7160424) variants used in this study were isolated from clinical specimen as previously described (23, 25).

## References

[1] Younes N, Al-Sadeq DW, Al-Jighefee H, Younes S, Al-Jamal O, Daas HI, Yassine HM, Nasrallah GK. 2020. Challenges in laboratory diagnosis of the novel coronavirus SARS-CoV-2. Viruses 12:582. doi:10.3390/v12060582. 32466458 PMC7354519

[2] Deerain J, Druce J, Tran T, Batty M, Yoga Y, Fennell M, Dwyer DE, Kok J, Williamson DA. 2022. Assessment of the analytical sensitivity of ten lateral flow devices against the SARS-CoV-2 omicron variant. J Clin Microbiol 60 jcm0247921. doi:10.1128/jcm.02479-21. PMC8849215 34936477

[3] Bekliz M, Perez-Rodriguez F, Puhach O, et al. 2022. Sensitivity of SARS-CoV-2 antigen-detecting rapid tests for Omicron variant. medRxiv: the preprint server for health sciences. doi:10.1101/2021.12.18.21268018. PMC9430749 35938792

[4] Kanjilal S, Chalise S, Shah AS, et al. 2022. Analytic sensitivity of the Abbott BinaxNOW lateral flow immunochromatographic assay for the SARS-CoV-2 Omicron variant. medRxiv: the preprint server for health sciences. doi:10.1101/2022.01.10.22269033.

[5] Metzger CMJA, Lienhard R, Seth-Smith HMB, Roloff T, Wegner F, Sieber J, Bel M, Greub G, Egli A. 2021. PCR performance in the SARS-CoV-2 Omicron variant of concern? Swiss Med Wkly 151:w30120. 34909869 10.4414/smw.2021.w30120

[6] Mak GC, Cheng PK, Lau SS, Wong KK, Lau CS, Lam ET, Chan RC, Tsang DN. 2020. Evaluation of rapid antigen test for detection of SARS-CoV-2 virus. J Clin Virol 129:104500. doi:10.1016/j.jcv.2020.104500. 32585619 PMC7278630

[7] Nordgren J, Sharma S, Olsson H, Jämtberg M, Falkeborn T, Svensson L, Hagbom M. 2021. SARS-CoV-2 rapid antigen test: high sensitivity to detect infectious virus. Clin Virol 140:104846. doi:10.1016/j.jcv.2021.104846. PMC8105081 33971580

[8] Pekosz A, Parvu V, Li M, Andrews JC, Manabe YC, Kodsi S, Gary DS, Roger-Dalbert C, Leitch J, Cooper CK. 2021. Antigen-based testing but not real-time polymerase chain reaction correlates with severe acute respiratory syndrome coronavirus 2 viral culture. Clinical Infectious Diseases: An Official Publication of the Infectious Diseases Society of America 73:e2861–e2866. doi:10.1093/cid/ciaa1706. 33479756 PMC7929138

[9] Smith RL, Gibson LL, Martinez PP, Ke R, Mirza A, Conte M, Gallagher N, Conte A, Wang L, Fredrickson R, Edmonson DC, Baughman ME, Chiu KK, Choi H, Jensen TW, Scardina KR, Bradley S, Gloss SL, Reinhart C, Yedetore J, Owens AN, Broach J, Barton B, Lazar P, Henness D, Young T, Dunnett A, Robinson ML, Mostafa HH, Pekosz A, Manabe YC, Heetderks WJ, McManus DD, Brooke CB. 2021. Longitudinal Assessment of Diagnostic Test Performance Over the Course of Acute SARS-CoV-2 Infection. J Infect Dis 224:976–982. doi:10.1093/infdis/jiab337. 34191025 PMC8448437

[10] Manabe YC, Sharfstein JS, Armstrong K. 2020. The need for more and better testing for COVID-19. JAMA 324:2153. doi:10.1001/jama.2020.21694. 33185688 PMC8162730

[11] Thomas E, Delabat S, Carattini YL, Andrews DM. 2021. SARS-CoV-2 and variant diagnostic testing approaches in the United States. Viruses 13:2492. doi:10.3390/v13122492. 34960762 PMC8703625

[12] FDA. Use of BD Veritor System for rapid detection of SARS-CoV-2 with the BD Veritor plus analyzer. 2021. https://www.fda.gov/media/139755/download.

[13] FDA. BinaxNOW™ COVID-19 Ag CARD. 2021. https://www.fda.gov/media/141570/download.

[14] FDA. InteliSwab COVID-19 rapid test—Instructions for use home test. 2022. https://www.fda.gov/media/149912/download.

[15] FDA. QuickVue SARS antigen test—Instructions for use. 2021. https://www.fda.gov/media/144668/download.

[16] Regan J, Flynn JP, Choudhary MC, et al. 2021. Detection of the omicron variant virus with the Abbott BinaxNow SARS-CoV-2 Rapid Antigen Assay. medRxiv 2021.12.22.21268219. 10.1093/ofid/ofac022 PMC8842316 35169591

[17] Hui KPY, Ho JCW, Cheung M-c, Ng K-c, Ching RHH, Lai K-l, Kam TT, Gu H, Sit K-Y, Hsin MKY, Au TWK, Poon LLM, Peiris M, Nicholls JM, Chan MCW. 2022. SARS-CoV-2 Omicron variant replication in human bronchus and lung ex vivo. Nature 603:715–720. doi:10.1038/s41586-022-04479-6. 35104836

[18] Government U. The SARS-CoV-2 variant, Omicron, shows enhanced replication in human primary nasal epithelial cells. https://assets.publishing.service.gov.uk/government/uploads/system/uploads/attachment_data/file/1043652/S1454_Omicron_report_20.12_Imperial.pdf.

[19] FDA. Bio-Rad SARS-CoV-2 ddPCR Test. 2020. https://www.fda.gov/media/137579/download.

[20] Gniazdowski V, Morris CP, Wohl S, et al. 2020. Repeat COVID-19 molecular testing: correlation of SARS-CoV-2 culture with molecular assays and cycle thresholds. Clin Infect Dis. 10.1093/cid/ciaa1616 PMC7665437 33104776

[21] Schaecher SR, Mackenzie JM, Pekosz A. 2007. The ORF7b protein of severe acute respiratory syndrome coronavirus (SARS-CoV) is expressed in virus-infected cells and incorporated into SARS-CoV particles. J Virol 81:718–731. doi:10.1128/JVI.01691-06. 17079322 PMC1797472

[22] Mostafa HH, Lamson DM, Uhteg K, Geahr M, Gluck L, de Cárdenas JNB, Morehead E, Forman M, Carroll KC, Hayden RT, George KS. 2020. Multicenter evaluation of the NeuMoDx SARS-CoV-2 Test. J Clin Virol 130:104583. doi:10.1016/j.jcv.2020.104583. 32791382 PMC7413157

[23] Fall A, Eldesouki RE, Sachithanandham J, et al. 2022. A quick displacement of the SARS-CoV-2 variant Delta with Omicron: unprecedented spike in COVID-19 cases associated with fewer admissions and comparable upper respiratory viral loads. medRxiv. 10.1016/j.ebiom.2022.104008 PMC9020587 35460989

[24] Morris CP, Luo CH, Amadi A, et al. 2021. An update on SARS-CoV-2 diversity in the United States national capital region: evolution of novel and variants of concern. Clin Infect Dis. 10.1093/cid/ciab636 PMC8406876 34272947

[25] Luo CH, Morris CP, Sachithanandham J, et al. 2021. Infection with the SARS-CoV-2 Delta variant is associated with higher recovery of infectious virus compared to the Alpha variant in both unvaccinated and vaccinated individuals. Clin Infect Dis. 10.1093/cid/ciab986 PMC8903351 34922338

[26] Thielen PM, Wohl S, Mehoke T, Ramakrishnan S, Kirsche M, Falade-Nwulia O, Trovão NS, Ernlund A, Howser C, Sadowski N, Morris CP, Hopkins M, Schwartz M, Fan Y, Gniazdowski V, Lessler J, Sauer L, Schatz MC, Evans JD, Ray SC, Timp W, Mostafa HH. 2021. Genomic diversity of SARS-CoV-2 during early introduction into the Baltimore-Washington metropolitan area. JCI Insight 6. doi:10.1172/jci.insight.144350. PMC8026189 33749660

[27] FDA. EUROIMMUN Anti-SARS-CoV-2 ELISA (IgG) Instruction for use. 2022. https://www.fda.gov/media/137609/download.

[28] Mostafa HH, Luo CH, Morris CP, et al. 2021. SARS-CoV-2 infections in mRNA Vaccinated Individuals are Biased for Viruses Encoding Spike E484K and associated with reduced infectious virus loads that correlate with respiratory antiviral IgG levels. medRxiv 2021.07.05.21259105. 10.1016/j.jcv.2022.105151 PMC8979609 35398602

